# Size effect characteristics and influences on fatigue behavior of laser powder bed fusion of thin wall GRCop-42 copper alloy^☆^

**DOI:** 10.1016/j.heliyon.2024.e28679

**Published:** 2024-03-27

**Authors:** Gabriel Demeneghi, Paul Gradl, Jason R. Mayeur, Kavan Hazeli

**Affiliations:** a NASA Marshall Space Flight Center, Huntsville, USA; b Mechanical and Aerospace Engineering Department, University of Alabama in Huntsville, USA; c Manufacturing Science Division, Oak Ridge National Laboratory, Oak Ridge, USA; d Mechanical and Aerospace Engineering Department, University of Arizona, USA

**Keywords:** Additive manufacturing, Thin wall, Size effects, Porosity, Tensile test, Fatigue

## Abstract

Size effects, influencing a material's strength, elongation, fatigue limit, and longevity, depend on the operative and dominant deformation and failure mechanisms. This study explores the size effects in additive manufactured (AM) GRCop-42 (Cu-4at%Cr-2at%Nb) thin wall structures fabricated via laser-powder bed fusion (L-PBF) and their impact on fatigue life. The influence of internal defects and surface topography on the fatigue life of specimens in both as-built and hot isostatic pressed (HIP) conditions across different thicknesses is investigated. Where micro-computed tomography (*μ*CT) was used to quantify the internal porosity of as-built, pristine HIP'd, and fatigued HIP'd specimens, and laser microscopy was employed to quantify the surface topography of specimens prior to fatigue. Additionally, quasi-static tests were used to establish baseline mechanical properties (i.e. yield strength (YS), ultimate tensile strength (UTS), and elongation) to frame fatigue testing conditions. Results indicate a significant enhancement in fatigue life for HIP'd specimens for both thicknesses, with internal defects depicting a greater impact than surface topography. Furthermore, fractographic analysis suggests that thicker specimens exhibit higher resistance to crack propagation during fatigue testing in the absence of substantial porosity. Thus, the size effects observed on the fatigue life of L-PBF GRCop-42 appears to be dominated by internal defects.

## 1 Introduction

GRCop-42 is a dispersion strengthened copper-chromium-niobium (Cu-4at.%Cr-2at.%Nb) alloy. GRcop alloys take advantage of the low solubility of chromium and niobium in the copper matrix and their high affinity for each other, forming a stable Cr_2_Nb intermetallic phase, which leaves an almost pure copper matrix, maintaining a high conductivity while simultaneously increasing the strength of the alloy. The precipitates provide strengthening by Orowan mechanisms hindering dislocation motion [1], [2], [3] and via the Hall-Petch mechanism by pinning the grains and thus preventing grain growth even at elevated temperatures [1], [2], [4], [5]. Due to GRCop-42 application in rocket combustion engine liners [6], [7], [8], [9], decreasing the wall thickness is necessary to maintain reasonable wall temperatures with the high fluxes from the environment, provided that it can sustain the in-service structural and thermal loads. Therefore, understanding the thickness effects on the mechanical properties is crucial for design safety and operation success.

Mechanical properties of materials are generally obtained from laboratory experiments performed on small specimens that serve as “witness” to components. This approach has been successful in a sense that it avoids the limitations that arise with testing full size components. However, the mechanical response of thin specimens cannot be assumed to be equal to bulk specimens [10], [11]. Therefore, it is important to understand how the size (i.e. thickness) of the specimen affects the material properties [12]. Generally speaking, the study of specimen size effects is divided into three main categories, (1) statistical size effects, which account for the probability of finding flaws within a region increases with the increase volume of the stressed region. (2) geometrical size effect, which accounts for changes in the stress gradient affects of fatigue life (i.e. decreasing the “notch” size decreases the fatigue life); and (3) technological size effects, which accounts for how changes in the processing method, which leads to changes in the component microstructure, surface topography, residual stresses, etc. [12], [13].

The fatigue strength of materials is related to the component size. It has been shown to decrease with increasing component size [13], [14], [15], [16]. This is a result of fatigue cracks initiating from mechanical discontinuities in the material from undetected flaws. Thus, increasing the volume of material in a component increases the probability of finding a critical-sized flaw. For example, EN-GJS-400-18-LT, a ductile cast iron used in wind turbine components, showed a higher fatigue strength for 21  diameter specimens than 50  diameter specimens. This was attributed to thicker blocks having lower cooling rates, and thus, lower nodularity and nodule size, decreasing the fatigue strength [16]. Additionally, Sun et al. [14] estimated that the fatigue strength for 90% survival probability of a full-scale railway axle (EA4T) is 33% lower than 4  specimens, at a fatigue life of 10^6^ cycles.

Although the specimen size effect was linked to a decrease in fatigue strength with increasing specimen size, the opposite trend has also been reported. Wang et al. [17] found that the fatigue strength of 18CrNiMo6-7 alloy steel increased as the diameter of the specimen increased from 3  to 7.5 , a possible reason for this behavior was the residual compressive stress on the surface of larger specimens was slightly larger than smaller specimens. Bu [18] observed that the crack propagation areas were the same for different sized specimens through fracture analysis of aviation ear flaps made from aluminum alloy, which means that the absolute length of fatigue cracks was longer for larger specimens. Additionally, it is important to note that the critical size of a defect increases with increasing component size [16].

Therefore, specimen size effects may lead to an increase or decrease in fatigue strength and life depending on the operative and dominant deformation and failure mechanisms for a particular material. Since components produced by AM typically contain internal defects (i.e. porosity, lack of fusion, etc.), increased surface roughness compared to wrought, microstructural heterogeneities, and residual stresses [19], [20], it would be rational to expect larger AM components to have shorter fatigue lives because of the increased probability of finding critical manufacturing induced defects. However, quasi-static testing of AM components showed a different trend [10], [11], suggesting different mechanics at play. Thus, technological size effects play a major role in determining the fatigue life of these components and the influence of these features need to be further understood.

Surface topography (form, waviness, roughness) of AM components has been shown to be a source of size effects in various studies. L-PBF Inconel 718 thin walls showed a significantly shorter high cycle fatigue (HCF) life for specimens with as printed surface when compared to machined specimens [21]. Another study on L-PBF Ti-6Al-4V showed that the as-printed surface roughness plays a key role in reducing the fatigue life in the low cycle fatigue (LCF) regime [22]. Similarly, surface roughness in L-PBF 304 L stainless steel (SS) [23] and L-PBF 316L SS [24] was shown to be the primary site for crack initiation during fatigue of specimens with as-printed surfaces. Moreover, defects at or near the surface were found to initiate cracks on machined and polished specimens. In addition to stress concentration and potential crack initiations, surface topography may lead to inaccuracies during cross-sectional area measurements. Thus, when determining the properties of specimens with varying sizes, it is important to accurately measure the effective cross-sectional area, as the surface roughness plays a major role in AM components. This has been demonstrated for L-PBF 316L SS [25], L-PBF 304L SS [26], electron beam melting Ti64 [26], and laser powder directed energy deposition GRCop-42 [11].

Internal defects and microstructure resulting from the AM process also play a role in the size effect. A study on L-PBF GRCop-42 [10] found that internal porosity was the leading cause of size effects during quasi-static testing as specimens' thickness decreased from 2  to 0.7 . L-PBF 316L tensile specimens had finer grains near the surface from the outer contour pass, which covered a larger percentage of the cross-sectional area for thinner specimens, leading to an increase in yield strength (YS) and a decrease in elongation with decreasing specimen width [27]. Additionally, the effect of surface roughness was shown to diminish compared to internal defects when a larger lack of fusion defects were present [28].

Additionally, it is worth mentioning that, defects resulting from AM fabrication methods can be remediated by post-fabrication through heat treatments and machining, where post-fabrication treatments such as HIP have been linked to the decrease of internal defects of AM parts [8], [29], [30], [31]. However, HIP has little effect on surface-connected pores or near-surface porosity [30], [31]. Further, it has been shown that HIP is ineffective in reducing porosity of fine structures such as thin wall structures and lattice structures [10], [32]. Furthermore, larger pores might be pressured into a flat morphology by HIP that could not be detected by uCT and be detrimental to fatigue life [19]. As a result, it is critical to investigate the benefits and limitations that HIP has on individual alloys and specimen designs.

This study focuses on the size effects on HCF of as-built and HIP'd GRCop-42. It aims systematically characterize the operative and dominant failure mechanisms present as the specimen thickness is reduced. Understanding the damage mechanism and damage accumulation during cyclic loading while loading limits remain in the elastic regime is crucial for various applications including heat exchanges, waveguides, and electronic components. It has been shown that HCF causes microscopic defects throughout the material due to a macroscopic elastic load. Such changes have the potential to alter the mechanical properties [33], [34]. Therefore, the influence of HCF on mechanical properties, porosity, and fractography is investigated for specimens of different thickness in both as-built and HIP conditions.

The article is divided into the following structure: section 2, Materials and Methods: contains the specimens fabrication, the equipment used, and the details on how the analyses were performed. Section 3, Results and Discussion: contains the findings and discussion for each test. Section 4, Summary: sums up the findings in a detailed summary for the reader.

## 2 Materials and methods

### 2.1 Specimen manufacturing

**Figure 1:**
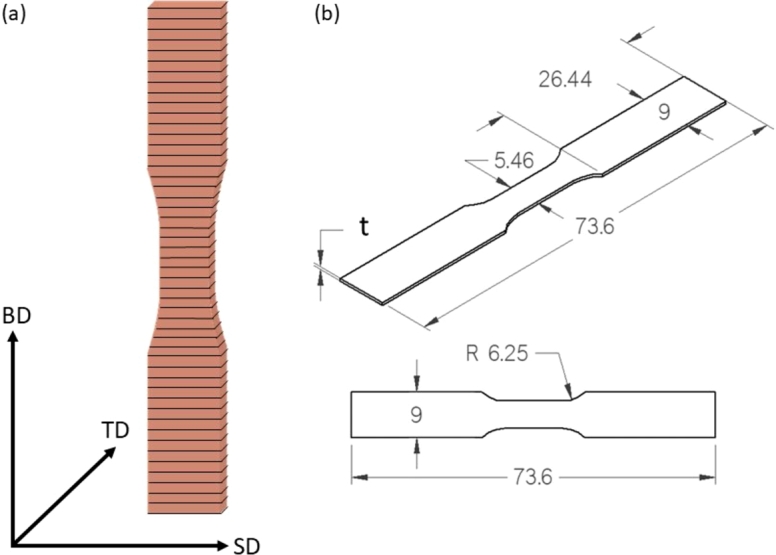
(a) Specimen build orientation relative to the build plate and (b) specimen dimensions. The same dimensions were used for both thicknesses, 1.5  and 2.1 , used in this study.

A total of 26 specimens were fabricated together in the same build as individual tensile flat bars parallel to the build direction (BD) using GRCop-42 pre-alloyed gas atomized powder. The specimens were fabricated via laser-powder bed fusion (L-PBF), which is a process where a thin layer of powder is spread over a vertically movable base plate inside of an ambient controlled chamber. A continuous wave laser then scans the pre-defined area based on the computer-aided design (CAD) file to melt the powder layer. Following this, the base plate moves down by one layer height and the process is repeated until the component is fully built [9], [35], [36]. The printer used was an EOS M400-1 Series printer with a Yb-fiber IR laser. The base plate was stainless steel coated with a nickel-based alloy (IN718). Specimens were fabricated with a power of 300 , 1000 , 0.04  layer thickness, and 0.1  hatching distance. A continuous scan pattern was used during the deposition process to prevent melt pool contamination with ejecta, which could be detrimental to mechanical properties due to possible lack of fusion defects [37], [38]. Additionally, a contour pattern was used to negate the effects the infill pattern has on the surface. Fig. 1 (a) shows a sketch of the specimen's build orientation relative to the build plate with the build direction, scanning direction (SD), and transverse direction (TD), and Fig. 1 (b) the specimen's dimensions, where the “*t*” represents the specimens' thickness as all other dimensions are held fixed. Two different thicknesses are considered, 2.1  and 1.5 . Where the 2.1  specimens show a comparable ultimate tensile strength (UTS) to standard size specimens, approximately 340  for HIP'd specimens [10], [39], while the 1.5  specimens show a decrease in YS, UTS, and elongation [10].

Following fabrication, specimens were removed from the build plate using electric discharge machining (EDM). Half of the specimens were sent for HIP, while the other half was retained in the as-built condition to establish the effectiveness of HIP on the specimens' mechanical response. Although the specific procedure for the HIP treatment is not disclosed due to proprietary data considerations, commonly applied procedures for copper alloys, as described in [40], were used.

### 2.2 Surface analysis

**Figure 2:**
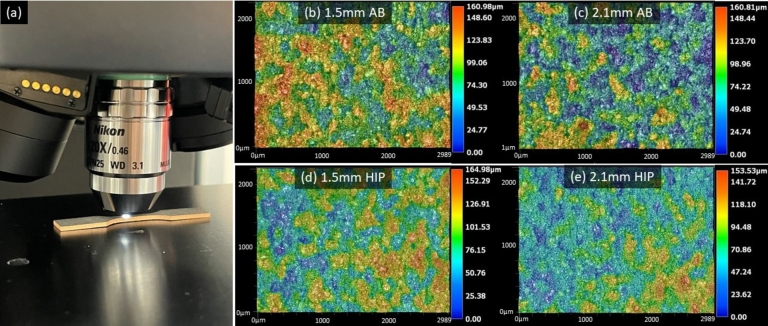
Representation of aerial surface topography measurements showing the maximum height of the surface (Sz). (a) Specimen being measured with Keyence VHX-1100 microscope, (b) 1.5  as-built specimen, (c) 2.1  as-built specimen dimensions, (d) 1.5  HIP'd specimen, and, (e) 2.1  HIP'd specimen. Although the surface topography appears smoother on HIP'd specimens due to the additional heat treatment, no appreciable quantitative difference is observed in measurements.

Surface analysis was performed using a Keyence VHX-1100 laser microscope equipped with a 20× magnification lens. Data were collected with a working distance of 3.1  using the widefield focus variation scan mode. The keyence VHX-1100 uses a 405  laser to map and measure the surface of specimens with a 20  vertical resolution. The aerial surface measurements were performed on the gage section of three specimens for each thickness and heat treatment condition. Three random locations with areas of approximately 6.5  were selected to perform the analysis. Fig. 2 (a) shows a specimen being measured with representative surface scans for (b) 1.5  as-built, (c) 2.1  as-built, (d) 1.5  HIP, and (e) 2.1  HIP specimens.

### 2.3 Porosity measurement

Porosity was measured using micro-computed tomography (*μ*CT) scanning technique as it is non-destructive, able to determine the shape and size of individual pores, and measures a statistically significant volume for each specimen [41], [42].

The analysis was performed on a Zeiss Versa 620 using 140  and 21  using a variable exposure scan mode to increase the scan time at longer path lengths; this decreases noise and reduces the scan time. A total of 1601 projections were generated for 180°rotation plus fan angle. A pixel size for the analysis was calculated to be 8.06 , resulting in a detectable porosity volume, voxel size, of around 526 . Each specimen was scanned along the gage section with depth and cross-section to commensurate with the specimen size. Table 1 shows the volume scanned, scan depth, and the average cross-sectional area measured with *μ*CT for the pristine specimens.

**Table 1:** *μ*CT measurements for 1.5 and 2.1  specimens in the as-built and HIP condition.

		Volume Scanned ()	Depth Scanned ()	Average Cross-Section Area ()
1.5	as-built	94.87	12.90	7.36
	HIP	82.48	11.67	7.07
2.1	as-built	142.78	13.30	10.74
	HIP	114.39	11.27	10.15

### 2.4 Quasi-static tensile tests

Quasi-static tensile testing was performed in air at ambient temperature using a servo-hydraulic materials testing system (MTS) machine equipped with a 100  load cell. The displacement rate was set to 0.5 , which corresponds to strain rates of $10^{-4}\;{\text{s}}^{-1}$. The strain was measured using digital image correlation (DIC) with Correlated Solution's Vic 2D software. The setup included a PointGrey Grasshopper camera (GS3-U3-51S5M-C) at a resolution of 2448×2048 px with a Cole-Parmer 41500-50 fiber optic illuminator. The collection frame rate during testing was set for 10 frames per second during the elastic deformation stage and 1 frame per second during plastic deformation. A subset size of 33 pixels with a step size of 11 pixels was used for the DIC analysis. Moreover, a standard deviation of the unloaded strain for all specimens was obtained from a series of images taken prior to loading the specimens and then calculated to be 127 *μϵ*.

### 2.5 Fatigue tests

Fatigue tests were run on air at ambient temperature in accordance with ASTM E466-21 on an Instron 8801 servo-hydraulic frame with a dynamic load capacity of ±100 , and actuator stroke of 150  positioned on the base and a frame stiffness of 390  equipped with a 97.86  fatigue rated load cell. Tests were initiated in load control at 30  with a stress ratio of R=0.1, and a sinusoidal waveform. Tests were run to failure or until 10^6^ cycles, whichever came first.

### 2.6 Microscopy

Optical and electron microscopy were used to assess the microstructure perpendicular to the fracture plane (BD-SD) and fracture surface (SD-TD) post tensile and fatigue testing. A LEICA DMi8 microscope running with Leica Application Suite X software was used to capture optical images. Specimens were hot resin mounted and polished down to 5  using SiC paper, then polished to 1  using a diamond solution, followed by a polished to a mirror-like finish using a 0.05  silica suspension. Directly following polishing, specimens were etched using a modified Kallings No 2 [43] (4  CuCl_2_, 80  of HCl, and 100  of ethyl alcohol) to reveal the grain structure. The specimens were submerged for approximately 30 s, wiped off with a cotton ball, rinsed with cold water for several seconds, rinsed in ethanol to remove water impurities, and then hot-air dried. Fracture surface images were captured using a Hitachi S-3700N scanning electron microscope (SEM). The SEM was operating at , with a probe current of , and a working distance of approximately 10 , in full vacuum mode.

## 3 Results and discussion

### 3.1 Surface analysis

**Figure 3:**
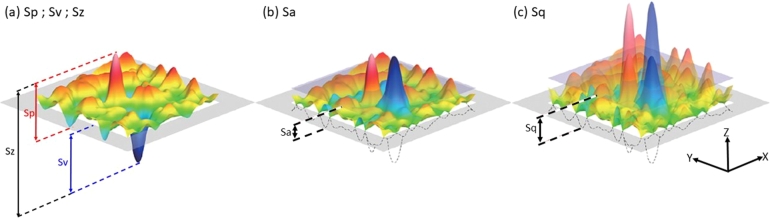
Graphical representation of the measure values for (a) Sp; Sv; Sz, (b) Sa, and (c) Sq. The gray plane represents the mean value for the measurements with indications of how each measurement is taken. Image courtesy of Evident Corporation.

In addition to measurement inaccuracies resulting from an uneven surface topography, fatigue cracks tend to initiate on the surface. Thus, it is necessary to characterize the overall surface topography of the specimens. Three locations were randomly selected in three different specimens for each condition to perform areal measurements and obtain metrics quantifying the surface topography. The following values were measured for surface characterization: average areal surface roughness (Sa), root mean square surface roughness (Sq), max measured valley depth (Sv), maximum measured peak height (Sp), and the collective range of maximum height of the surface (Sz). Fig. 3 shows representative images of how the surface topography values for (a) Sp, Sv, and Sz, (b) Sa, and (c) Sq are obtained.

Table 2 shows the measured values of Sa, Sq, Sv, Sp, and Sz. These are calculated using the following equations with the peak height *Z* as a function of the coordinates *x* and *y*, respectively:

(1) $$Sa=\frac{1}{A}\iint_{A}|Z(x,y)|dxdy$$

(2) $$Sq=\sqrt{\frac{1}{A}\iint_{A}Z^{2}(x,y)dxdy}$$

(3) Sv=|min(Z(x,y))|

(4) Sp=|max(Z(x,y))|

(5) Sz=Sp+Sv

**Table 2:** Measured surface topography values obtained for both thicknesses and heat treatment conditions.

		Sa	Sq	Sv	Sp	Sz
1.5 mm	as-built	19.9 ±1.5	24.8 ±1.8	79.4 ±8.7	90.3 ±10.3	169.6 ±12.3
	HIP	20.0 ±2.8	25.1 ±3.3	74.5 ±7.9	90.7 ±14.6	165.2 ±18.2
2.1 mm	as-built	20.9 ±2.3	26.0 ±2.5	76.7 ±5.3	83.7 ±8.3	160.4 ±10.8
	HIP	20.3 ±2.0	25.2 ±2.3	73.2 ±10.5	89.2 ±9.0	162.4 ±17.1

Specimens display similar values for all measured surface topographic features, indicating that the surface topography of the AM specimens does not vary with specimen thickness or heat treatment conditions. It has been suggested that stress concentrations resulting from the surface roughness of specimens lead to a decrease in YS, UTS, elongation, and Young's Modulus [25], [26], [44], and that the magnitude of the stress concentration also depends on the magnitude of the surface roughness [45]. Therefore, the present surface roughness on these specimens may not be sufficiently large to cause significant stress concentration. However, it can still lead to inaccurate cross-sectional areas if using contact devices, such as calipers, and need to be accounted for. Furthermore, specimens with different thicknesses but a similar surface roughness and width implies that surface roughness occupies a larger percentage of the caliper measured cross-sectional area as the specimen thickness is reduced. Where and approximate cross-sectional area reduction of 25% and 20% is observed for 1.5  and 2.1  specimens, respectively.

### 3.2 Quasi-static tests

Quasi-static tensile tests were performed to obtain mechanical properties such as YS, UTS, and elongation, which were used to determine appropriate fatigue testing conditions. Two specimens for each thickness and HIP condition were loaded to failure to study the size effects on the mechanical response. Since the authors have previously reported the quasi-static behavior of L-PBF GRCop-42 [10], only two specimens were needed to confirm that specimens were following the previously reported measurements. It is important to note that although the printers and the process parameters (laser powder and scan speed) were different for both builds, the energy density was similar (approximately 75 ). Additionally, the tested specimen displayed similar trends of decreasing strength and elongation with decreasing thickness, eluding to these size effects not being only dependent on the printer and specific process parameters.

**Figure 4:**
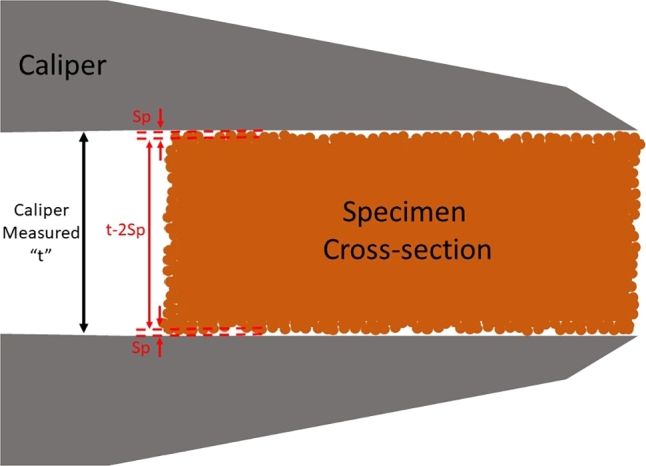
Schematic of an exaggerated L-PBF specimen's cross-sectional area being measured using calipers. Note that calipers make contact with the higher peaks on the surface of the specimen. Thus, removing the *Sp* from each as-printed surface removes the highest peaks measured, approximating the value to the mean surface plane.

As it was discussed in detail [10], measurement of the load-bearing cross-sectional area can be significantly affected by the surface roughness and partially melted or powder adhered to the surface, therefore this must be accounted for. Accordingly, the maximum peak height, *Sp*, was subtracted from the caliper measured dimensions because it represents the highest peaks on the surface, and thus, it is likely where a caliper will make contact first. Fig. 4 shows a sketch of a L-PBF specimen cross-section being measured with calipers, note that the calipers make contact with the peaks of the surface, which do not necessarily represent the load-bearing area of the specimen.

The stress is calculated with the following equation:

(6) $$\sigma_{c}=\frac{F}{A_{c}}$$

where $A_{c}$ is calibrated area defined as:

(7) $$A_{c}=(w-2Sp)\times (t-2Sp)$$

Where $\sigma_{c}$ is the calibrated stress, *F* is the force, *w* is the caliper measured width, *t* is the caliper measured thickness, and *Sp* is the maximum peak height. Twice the *Sp* value is subtracted from both areal dimensions to properly account for specimen symmetry. Previous works arrived at similar conclusions, for example, Yu et al. [46] subtracted twice the maximum profile peak height (Rp) from the thickness prior to multiplying by the width to correct for the surface roughness. Variants of these corrective methods have been presented in other studies, cf. [11], [47].

**Figure 5:**
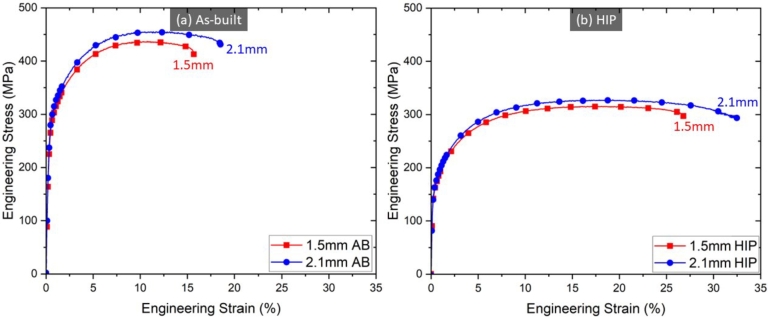
Quasi-static results for (a) as-built and (b) HIP specimens for both thicknesses. A distinct size effect between the 1.5  and 2.1  specimens is observed, with the thicker specimen having a higher elongation and strength when compared to the thinner specimen. Additionally, HIP lowered the strength of specimens for both thicknesses but increase the total elongation prior to failure.

**Figure 6:**
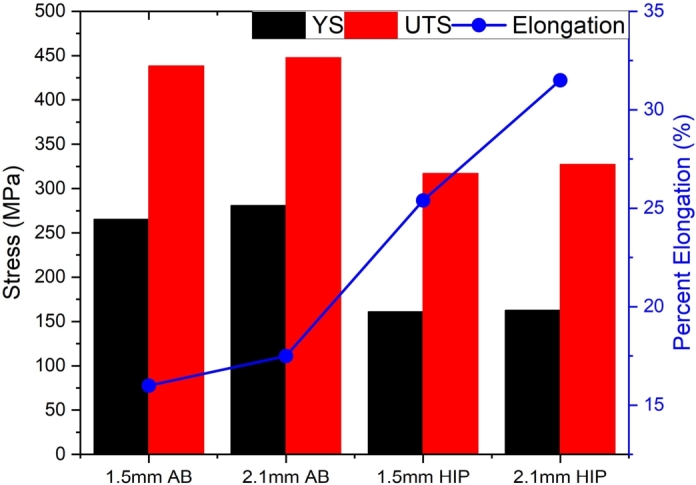
YS, UTS, and elongation comparison for 1.5  and 2.1  specimens in the as-built and HIP'd conditions.

Fig. 5 shows the engineering stress-strain curve for both thicknesses in the (a) as-built and (b) HIP'd conditions, and Fig. 6 displays a bar chart for the YS, UTS, and elongation for 1.5  and 2.1  specimens in the same conditions. A clear size effect is observed for both as-built and HIP'd specimens, indicating that the size effect is either independent or not fully eliminated by the heat treatment.

Compared to the as-built specimens, a significant decrease in YS and UTS is observed on all HIP'd specimens. 2.1  specimen had a 42% decrease in YS and a 27% decrease in UTS specimen compared to a 39% decrease in YS and a 28% decrease in UTS for 1.5  specimen. This behavior is expected and attributed to relieving residual stresses in L-PBF metals [48], which could be detrimental to the mechanical properties [49]. On the other hand, elongation increases for specimens in the HIP'd condition, with a 59% elongation increase for 1.5  specimen and a 80% elongation increase for 2.1  specimen. HIP has been shown to positively impact the elongation of IN718, improving it by 20% when compared to as-built specimens [9], [21].

HIP also led to a slight change in the magnitude difference in strength and elongation for both thicknesses. For specimens in the as-built condition, YS, UTS, and elongation was 6%, 2%, and 9% higher for 2.1  specimen compared to 1.5  specimen, respectively. HIP'd specimens showed a 1% higher YS, 3% higher UTS, and 19% higher elongation for 2.1  compared to 1.5  specimens. While the difference in strength between the two thicknesses is relatively small, the difference in elongation is significantly larger for HIP'd specimens. The increase in elongation is presumed to be due to the porosity reduction post HIP since the elongation to failure has been observed to increase with increasing thickness for GRCop-42 [10] and IN718 [21].

Table 3 shows the Young's Modulus, YS, UTS, and percent elongation for 1.5  and 2.1  specimens in both as-built and HIP'd conditions.

**Table 3:** Average mechanical properties obtained from the quasi-static test.

	Condition	Young's Modulus (GPa)	YS (MPa)	UTS (MPa)	Elongation (%)
1.5	As-built	96.1 ±3.3	265.5 ±0.0	438.4 ±1.2	16.0 ±0.3
	HIP	82.7 ±2.0	161.0 ±0.8	317.1 ±1.8	25.4 ±1.3
2.1	As-built	82.4 ±1.1	281.0 ±0.8	447.9 ±1.0	17.5 ±1.7
	HIP	94.5 ±1.7	162.8 ±0.0	327.2 ±0.4	31.5 ±0.0

A decrease in strength, YS and UTS, and elongation are observed as the thickness of the specimen decreases from 2.1  to 1.5  in both as-built and HIP'd conditions. A decrease in YS, UTS, elongation, and Young's Modulus was previously observed in L-PBF 316L stainless steel [25], L-PBF 304L stainless steel [26], and electron beam melting Ti64 [26], which were attributed to the surface roughness of the specimen, causing stress concentration and a reduction on the load bearing area. GRCop-42 also showed a reduced strength and elongation with decreasing specimen size [10], however, for GRCop-42, the decrease in strength and elongation is attributed to increased internal defects as the specimen thickness decreased. Porosity has been shown to be detrimental to the mechanical properties of AM Ti-6Al-4V [50], [51], AM stainless steels [52], and others [53].

### 3.3 Fatigue tests

The effects of specimen size and heat treatment condition regarding fatigue life were investigated in a load-controlled tensile-tensile (R=0.1) environment, at ambient temperature and air. Eight specimens for each thickness were loaded along the BD axis in both as-built and HIP'd condition, four specimens for each. Tests were performed to failure or up to 10^6^ cycles with $\sigma_{max}=\frac{2}{3}\sigma_{y}$, where $\sigma_{y}$ was determined as the 0.2% offset from the tensile tests shown in section 3.2.

Table 4 summarizes the results of the fatigue tests for each thickness, condition, cycles to failure, and applied stress. Note that the applied stress, since it is defined in terms of the YS, varied for the different thickness/condition combinations.

**Table 4:** HCF life cycles for 1.5  and 2.1  specimens in both conditions, as-built and HIP'd. Both thicknesses show that HIP'd specimens did not fail before 10^6^ cycles, while as-built specimens failed at significantly lower cycles.

Specimen	Condition	Cycles	Stress ()
1.5	as-built	125,014	175
	as-built	71,493	175
	as-built	79,964	175
	as-built	119,105	175
	HIP	1,000,000	104
	HIP	1,000,000	104
	HIP	1,000,000	104
	HIP	1,000,000	104

2.1	as-built	101,172	193
	as-built	111,321	193
	as-built	99,668	193
	as-built	97,085	193
	HIP	1,000,000	110
	HIP	1,000,000	110
	HIP	1,000,000	110
	HIP	1,000,000	110

As-built 1.5  specimens failed at an average of 98,894 ±23,421 cycles exhibiting larger scatter when compared to as-built 2.1  specimens, which failed between 102,312 ±5,403 cycles. HIP'd specimens were tested to 10^6^ cycles without failure independent of the thickness.

**Figure 7:**
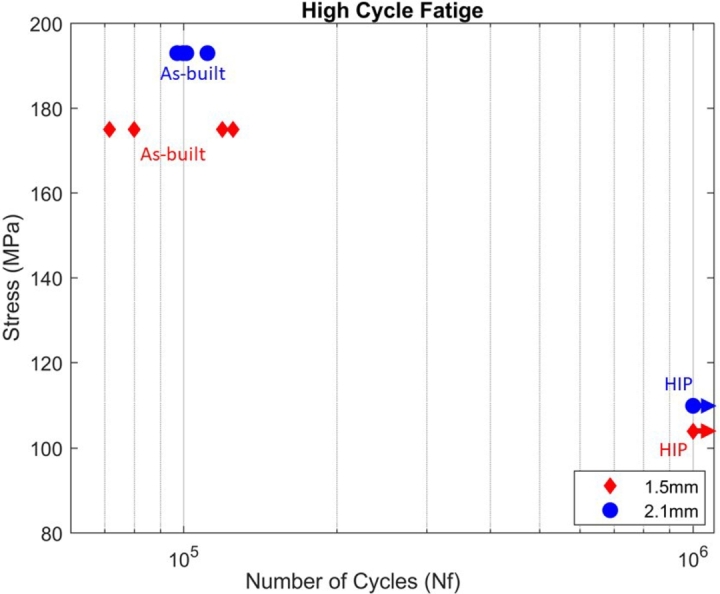
S-N curves for (a) 1.5  and (b) 2.1  specimens in the as-built and HIP'd conditions. As-built specimens failed at approximately 10^5^ cycles for both 1.5  and 2.1  specimens while specimens that were HIP'd went to 10^6^ before the test was stopped.

Fig. 7 shows the S-N curve for all fatigue tests performed. Note that the amount of scatter was larger for thinner specimens.

Both thicknesses showed relatively consistent cycles to failure for the as-built and HIP'd specimens, with HIP'd specimens showing a significantly higher fatigue life than as-built specimens. This indicates that HIP'ing greatly enhances the fatigue life of L-PBF GRCop-42 due to its effects on eliminating internal porosity. Porosity has been repeatedly linked to a decrease in fatigue life of AM alloys [19], [21], [54], [55], [56]. For instance, selective laser melting (SLM) AlSi_10_Mg, where the improvement of fatigue strength was attributed to a 64% decrease in porosity and the formation of intermetallic phases, improving the resistance to fatigue crack growth [56], and also in IN718 [21], where it was found that a lack of voids in the specimens delayed the fracture, leading to increased elongation in HIP'd specimens when compared to as-built specimens.

Furthermore, for AM alloys, it has been reported that surface roughness plays a larger role in the fatigue strength for as-built specimens, as, generally, near surface defects have higher stress concentrations than internal defects [20]. However, once specimens are machined, cracks are seen to initiate within internal defects [57]. Other material attributes of AM alloys, such as microstructural inhomogeneities, residual stresses, and anisotropy have a more substantial influence on response once the effects of surface roughness and internal defects are minimized [19].

Since all specimens had an as-printed surface, i.e., no surface finishing steps were applied, it is presumed that internal defects have a greater contribution to the decrease in the fatigue life than the surface condition. This is because HIP'd specimens had a reduction in internal defects, which led to an extended fatigue life. Additionally, as both thicknesses experienced a similar cycle to failure at stresses around 66% for their respective YS, it is presumed that at an equal stress level, thicker specimens would have a longer fatigue life than thinner specimens since under the same stress amplitude the crack propagation rate would be the same for specimens of different thickness, thus, thicker specimens would have longer crack propagation lives than thinner specimens [16].

### 3.4 Porosity measurements

To better understand the role of internal defects on damage evolution and crack propagation, the porosity was measured in pristine samples for both thicknesses and conditions through *μ*CT scans. Additionally, porosity was measured for one specimen for each thickness in the HIP'd condition after being fatigued to investigate the evolution of porosity distribution during cyclic loading. It is important to note that specimens were *μ*CT scanned prior to having detectable cracks forming in them to avoid interference by the fracture surface or micro-cracking. Table 5 shows the scanned volume, the total void volume, and the percent porosity for both 1.5  and 2.1  specimens in the as-built pristine, HIP pristine, and HIP fatigued.

**Table 5:** Shows the total scanned volume, total void volume, and calculated porosity percentage for specimens in each thickness and condition.

	Condition	Total Volume ()	Void Volume ()	Porosity (%)
1.5 mm	as-built	94.87	0.19	0.20
	HIP pristine	82.48	0.07	0.08
	HIP fatigued	97.76	0.03	0.03

2.1 mm	as-built	142.78	0.08	0.06
	HIP pristine	114.39	0.06	0.05
	HIP fatigued	151.49	0.02	0.01

**Figure 8:**
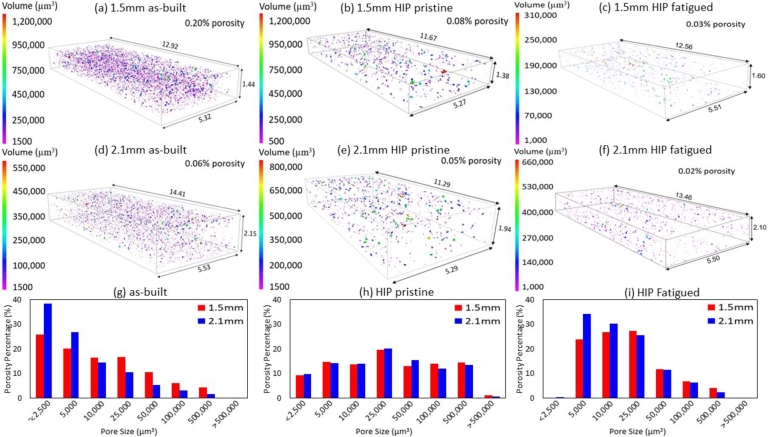
3D *μ*CT data reconstruction for the porosity analysis and the number of pores with their respective pore volume for (a)(d)(g) as-built specimens, (b)(e)(h) HIP'd pristine specimens prior to fatigue, and (c)(f)(i) HIP'd specimens post fatigue. HIP'd pristine is shown to significantly reduce the porosity percentage compared to as-built specimens, especially small pores. However, on HIP'd post fatigue specimens, small and medium pore percentages increased, with fewer larger pores.

The overall porosity percentage is higher for thinner specimens regardless of the heat treatment condition. In the as-built condition, the porosity percentage in 1.5  specimen is over three times that of the 2.1  specimen. HIP'd specimens show a decrease in porosity for both specimens, with a greater impact on thinner specimens. However, 1.5  specimens still have a higher porosity percentage than the 2.1  specimens. HIP'ing has a clear benefit in closing out internal porosity, shown by decreasing the volume percentage on HIP'd specimens for both thicknesses compared to its as-built specimens counterparts. This has been extensively shown in prior works [10], [29], [58]. Fig. 8 shows the *μ*CT scans 3D reconstruction and bar charts with the porosity percentage and their respective volumetric pore size for specimens in the as-built pristine, HIP'd pristine and HIP'd fatigued. Fig. 8 parts (a)(d)(g) display the porosity results for as-built specimens, Fig. 8 parts (b)(e)(h) show porosity in HIP'd pristine specimens, and Fig. 8 parts (c)(f)(i) shows the porosity results for HIP'd specimens after following fatigue testing to 1,000,000 cycles.

For specimens in the as-built condition, the percentage of smaller pores is higher for 2.1  specimens (up to 5,000 ), however, the percentage of pores larger than 5,000  is higher in 1.5  specimens. In HIP'd pristine specimens, the pore percentage is similar for both thicknesses for their respective void volume. A more significant decrease in smaller pores compared to larger pores, meaning that HIP is more effective in closing smaller pores (<25,000 ). These results are consistent with a previously published paper on size effects of GRCop-42 [10], where HIP showed little effect on closing out larger pores. On HIP'd fatigued specimens, the percentage of smaller pores (between 2,500  to 25,000 ) is higher when compared to HIP'd pristine specimens. However, pores larger than 25,000  experience a progressive decay when compared to HIP'd pristine specimens. Additionally, 2.1  specimens show a higher percentage of small pores (< 10,000 ) compared to 1.5  specimens, while pores larger than 10,000  are higher on 1.5  specimens.

**Figure 9:**
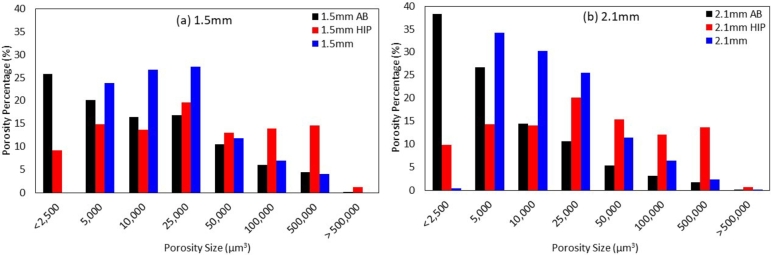
Porosity percent with respect to pore volume for (a) 1.5  and (b) 2.1  specimens in the pristine as-built condition, pristine HIP'd condition, and fatigued HIP'd condition.

Fig. 9 shows the percent porosity found for each pore volume for (a) 1.5  and (b) 2.1  specimens in the as-built condition, pristine HIP'd condition, and fatigued HIP'd condition. The near absence of pores smaller than 2,500  and the increase in pores between 5,000 and 25,000  post fatigue suggest void coalescence. The progressive decrease in the percentage of larger pores, greater than 25,000 , is likely an artifact of the increase in smaller pores since there was a 30% and 43% increase for pores smaller than 25,000  for fatigued 1.5  and 2.1  specimens when compared to the respective pristine specimens, thus, reducing the percent of larger pores. HCF of *α*-iron at a constant stress amplitude of 70% of the YS showed pore growth was more likely than pore formation, where the pore size increased while the overall pore ratio remained relatively the same for specimens that were cycled between 37%-73% of the fatigue life [33]. However, HCF of Aluminum 7075-T6 at tensile means stresses of 0  and 194  for 75% of the fatigue life lead to pore formation instead of growth, which was attributed to the increase in pores with an area of 10  while no significant pores larger than that were found [34].

**Figure 10:**
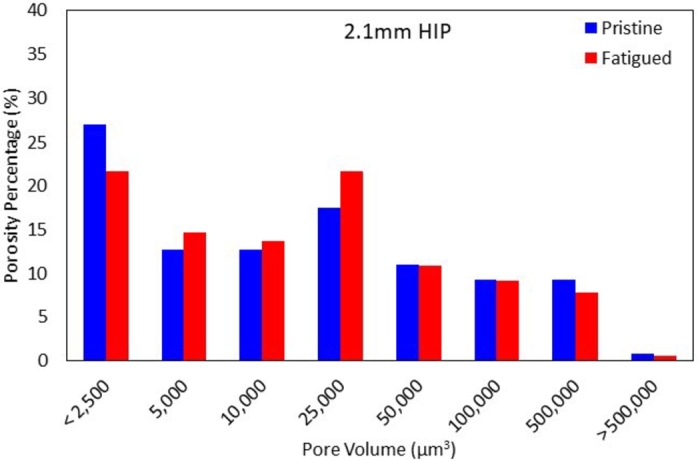
Porosity percentage with respect to the pore volume found on a HIP'd 2.1  specimen before and after fatigue testing to 10^6^ cycles. Porosity increases for pores larger than 2500  up to 25 000  for fatigued specimens and a similar porosity percentage or slight decay for pores larger than 25 000 .

In order to obtain a direct correlation between fatigue cycling on the internal porosity, two additional 2.1  specimens were used to obtain porosity using *μ*CT scanning, fatigued to 10^6^ cycles at 110  (two-thirds of the YS). The porosity of these specimens was then measured using *μ*CT scanning at the identical location. Fig. 10 shows the porosity percent found on 2.1  HIP'd specimen in the pristine condition and post fatigue. The specimen followed these steps: (1) *μ*CT - (2) HCF - (3) *μ*CT.

Similar to the results shown previously, the porosity percentage increases for pores with sizes between 2,500  and 25,000 , while pores larger than 25,000  show a similar, or slightly larger pore percentage for pristine specimens. The trend indicates that smaller pores decreased while larger pores increased or remained unchanged after fatigue. Analyzing the total number of pores in the specimen, a reduction in porosity is observed for all pore size ranges as well as the total pore volume, from 0.07  to 0.04  for the pristine and tested specimen, respectively. However, a larger decrease in pores smaller than 2,500  (≈48%) was observed, indicating that pore growth was more likely than new pore nucleation.

#### 3.4.1 Quasi-static test post fatigue

**Figure 11:**
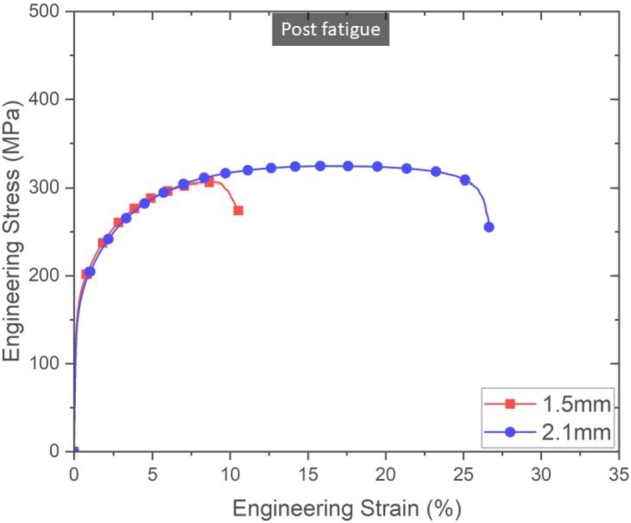
Quasi-static response of 1.5  and 2.1  HIP specimens after 10^6^ cycles. Although both specimens appear to have a decrease in elongation when compared to pristine specimens, the 1.5  specimen shows a significantly lower elongation than the 2.1  specimen.

Pre-existing fatigue history of a component has been shown to influence the mechanical properties, such as YS, UTS, and elongation [33], [34], [59]. However, the mechanical response of fatigued specimens cannot be readily generalized, as the mechanisms by which a material yields and fails are not the same as the ones in fatigue. Furthermore, the microstructure, internal defects, and surface roughness influence fatigue differently than YS and ductility. Therefore, the damage accumulated during cyclic loading was investigated through quasi-static tests of fatigued specimens. One fatigued specimen for each thickness, 1.5  and 2.1 , in the HIP condition was tested to investigate changes in mechanical properties as a consequence of the fatigue loading. Fig. 11 shows the engineering stress-strain response of the 1.5  and 2.1  HIP'd specimens after being subjected to 10^6^ cycles.

Although little change (less than 5%) in the strength was observed for both specimens, there was a considerable reduction in elongation for both HIP'd specimens. 1.5  specimen had a 61% reduction in elongation, going from 27% to 11% and 2.1  specimen had a 17% reduction in elongation, going from 32% to 27%. This is evidence of damage accumulation during fatigue testing, which effectively reduced the ductility of the specimens.

The decrease in elongation for the thinner specimen after fatigue eludes to a larger damage accumulation during cyclic loading for thinner specimens. Indeck et al. [33] found that the YS, UTS, and ductility of *α*-iron during quasi-static testing decreased by approximately 20% after fatigue cycling to 31%-94% of the fatigue life. Aluminum 7075-T6 experienced a 7% decrease in strength after 75% of the fatigue life, at mean stress of 194 , which was attributed to fatigue induced porosity, however, the elongation did not change significantly due to fatigue loading [34].

### 3.5 Fractography

Fractography was performed on specimens post tensile and fatigue testing. The specimens were optically imaged perpendicular to the fracture plane to investigate damage accumulation in the gage section. Additionally, fractographic analysis was conducted on the fracture surface through SEM to identify features that indicate ductile and brittle fractures.

**Figure 12:**
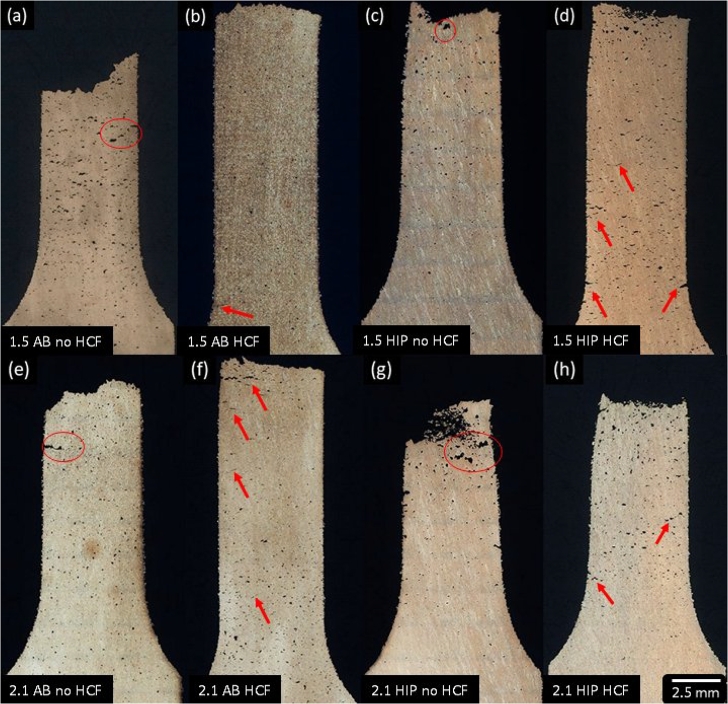
Fractured specimens perpendicular to the fracture plane for (a)(b)(c)(d) 1.5  and (d)(f)(g)(h) 2.1  specimens. (a) and (e) As-built specimens with no HCF, (b) and (f) as-built specimens + HCF, (c) and (g) HIP'd specimens with no HCF, (d) and (h) HIP'd specimens + HCF. Red circles highlight pore growth and coalescing, and red arrows point to secondary cracks and voids coalescing.

Fig. 12 shows the fractured specimens perpendicular to the fracture plane for both 1.5  and 2.1  specimens in the as-built and HIP'd condition as well as a comparison between fatigued specimens and quasi-static tested specimens. Fig. 12 parts (a), (b), (c), and (d) show (a) 1.5  as-built specimen tensile tested, (b) 1.5  as-built specimen HCF tested, (c) 1.5  HIP'd specimen tensile tested, and (d) 1.5  HIP'd specimen HCF tested. While Fig. 12 parts (e), (f), (g), and (h) show (e) 2.1  as-built specimen tensile tested, (f) 2.1  as-built specimen HCF tested, (g) 2.1  HIP'd specimen tensile tested, and (h) 2.1  HIP'd specimen HCF tested. Fatigued specimens showed a relatively flat fracture plane when observed perpendicular to the fractured plane in both as-built and HIP conditions. Additionally, cracks were nucleating and growing perpendicular to the load direction along the width of the gage section, while quasi-static tests on pristine specimens lead to slanted fracture surfaces with shear lips visible. Void coalescence was also observed throughout the gage section, especially near the fracture surface for both fatigued and pristine specimens. Seltzman et al. [3] found that GRCop-84 tested under quasi-static loading failed due to void nucleation originating on the fractured Cr_2_Nb intermetallic phase in the copper matrix by identifying Cr_2_Nb precipitates inside over 80% of the ductile dimples in the fracture surface. Therefore, near surface defects likely play a larger role in the fatigue life than void nucleation from fractured Cr_2_Nb particles.

**Figure 13:**
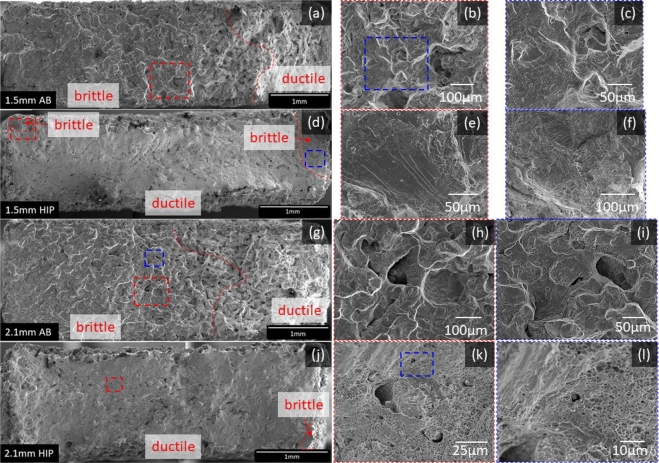
Fracture surface for 1.5  and 2.1  specimens in both as-built and HIP'd conditions. (a) shows the 1.5  as-built fracture surface with a zoomed in version at (b) showing unmelted powder and a further zoomed in image (c) showing striation marks. Part (d) shows the 1.5  HIP'd fracture surface with zoomed in sections at (e) and (f) to show brittle fracture starting at the corners of the specimen. Part (g) shows the 2.1  as-built fracture surface with two zoomed in locations at (h) and (i) to show unmelted powder and striation marks, respectively. Part (j) shows the 2.1  HIP'd fracture surface with a zoomed in section (k) showing a defect surrounded by ductile dimples and a further zoomed in image (l) to show a debonded particle from the matrix.

Fig. 13 shows the fracture surface for 1.5  and 2.1  specimens in both as-built and HIP'd conditions. Where as-built specimens were fatigued until failure and HIP'd specimens were fatigued to 10^6^ cycles and then quasi-static tested to failure. Part (a) shows 1.5  as-built fracture surface with a zoomed in sections at parts (b) and (c). Part (d) shows the 1.5  HIP'd specimen with zoomed in sections at parts (e) and (f). Part (g) shows 2.1  as-built specimen with two zoomed in locations at parts (h) and (i). And part (j) shows 2.1  HIP'd specimen with zoomed in sections at parts (k) and (l).

Although all fractured specimens appear to have the fracture starting at or near the surface, the fracture surfaces for as-built and HIP show significant differences. As-built specimens showed over three-quarters of the fracture surface with brittle fracture, left to right in Fig. 13 (a), and overloaded the remaining area, characterized by a duller fracture surface. Additionally, striation marks, which are evidence of cyclic loading, were observed on the as-built specimens, and internal porosity and unmelted powder were more readily observed on as-built specimens (Fig. 13 (b)(c)(h)(i)). HIP'd specimens in Fig. 13 (d)(j), on the other hand, showed brittle features on corners, such as cleavage fracture, but ductile dimples characterized the majority of the surface from the overload during the subsequent quasi-static test. Furthermore, particles were found debonded from the matrix (13 (l)), which could lead to porosity formation [3].

A brittle fracture also composed a larger area of the fracture surface for 1.5  specimens compared to 2.1  as-built and HIP'd specimens. Wang et al. [17] found that the fatigue propagation area in specimens with different diameter sizes (3 , 5 , and 7.5 ) accounted for approximately 50% of the fracture surface, thus, the crack propagation area was larger on thicker specimens than thinner specimens, Wang et al. related this phenomenon back to the Paris equation, which states that under the same stress amplitude, the crack propagation should be the same for specimens of different thicknesses. Additionally, it was observed that HCF life increased with increasing specimen thickness for HIP'd thin-walled In718 [21]. Thus, thicker specimens possess a higher fracture toughness and delay the onset of the fracture.

Finally, fractures appear to start on the corners of the cross-sectional area, it is presumed that cracks tend to form on pores near the surface or surface defects of the specimens. This is unsurprising as defects near the surface produce higher stress concentrations than internal defects [20]. Furthermore, crack propagation appears to be the primary contributor to the fatigue failure of the specimens. It has been shown that pores with local curvatures smaller than 10  leads to crack initiation in tens of cycles [60]. Therefore, it is presumed that cracks would initiate early in the fatigue life and propagate until failure.

## 4 Summary

This study presents specimen size effects and damage accumulation observed on pristine and fatigued specimens of two different thicknesses and two heat treatment conditions, as-built and HIP'd, for L-PBF GRCop-42. The effect of HIP on fatigue life and damage accumulation during fatigue testing on HIP'd specimens were investigated through quasi-static loading, HCF, *μ*CT scanning, and microscopy. The findings are listed below:

• Surface topography was similar for all fabricated specimens regardless of specimen thickness or heat treatment condition. Although a similar surface topography for all specimens means that a smaller portion of the measured cross-section act as a load bearing area, fatigue life does not appear to be significantly dependent on surface topography. This was concluded based on both specimen thicknesses having a similar fatigue life at their given stress level.
• Quasi-static loading of as-built and HIP'd specimens show a clear dependence on the specimen thickness where the strength and elongation of the specimens decrease with decreasing thickness.
• As-built specimens for both thicknesses failed around 10^5^ cycles, while HIP'd specimens reached run out at 10^6^ cycles with no failures. Therefore, fatigue life was greatly enhanced by HIP'ing specimens, due to a decrease in internal porosity post HIP.
• Porosity was highest for the as-built specimen, with most pores being smaller than 25,000  for both thicknesses. Porosity decreased significantly with HIP, which was more effective in closing smaller than larger pores. Post fatigue, specimens showed a trend of increasing small and medium pore percentages, between 2,500  and 25,000 . Although the number of pores decreased for all pore sizes after fatigue, a greater decrease was observed in pores smaller than 2,500 . This indicates that pores are more likely to grow than to nucleate new pores.
• Quasi-static testing post fatigue showed decreased elongation for both specimen thicknesses. Where 2.1  specimens had a 17% reduction in elongation and 1.5  specimens had over 60% reduction in elongation. This indicates that 1.5  specimens accumulated larger damage during fatigue testing. Fractography confirms this observation by having a larger porting of the cross-section displaying a brittle fracture. However, no statistical significance can be attributed to this test as only one specimen of each thickness was quasi-statically tested post fatigue.
• The fracture surface of as-built fatigued specimens had a brittle fracture covering over half of the fracture surface and significant porosity throughout the fracture surface for both thicknesses. HIP'd specimens, that were fatigued to 10^6^ cycles and then quasi-static tested to failure, showed fracture initiation on the corners, which was presumed to have started during fatigue testing as it has a brittle nature, and contained minimal porosity on the fracture surface.

These results have significant implications on the application of AM thin structures as reducing the component size (i.e. thickness) can affect its life and performance. Properly determining the effective loading bearing area and using the appropriate post-fabrication process on AM components have severe consequences on determining material properties that are used in designs. Finally, as component reusability is becoming more common, understanding how the pre-existing fatigue history impacts the material properties is crucial for the safe implementation of new and used hardware.

## CRediT authorship contribution statement

**Gabriel Demeneghi:** Writing – original draft, Methodology, Formal analysis, Data curation, Conceptualization. **Paul Gradl:** Writing – review & editing, Validation, Supervision, Project administration, Methodology. **Jason R. Mayeur:** Writing – review & editing, Writing – original draft, Validation, Supervision, Investigation. **Kavan Hazeli:** Writing – review & editing, Validation, Supervision, Methodology, Investigation, Conceptualization.

## Declaration of Competing Interest

The authors declare that they have no known competing financial interests or personal relationships that could have appeared to influence the work reported in this paper.

## Data Availability

Data will be made available on request.
